# Efficacy and Safety of Docetaxel Plus Oxaliplatin and Capecitabine in the First Line Treatment of Advanced Gastric Adenocarcinoma

**DOI:** 10.1155/2013/971096

**Published:** 2013-09-15

**Authors:** Ying Liu, Zhengbao Ye, Wenqi Xi, Tao Ma, Min Shi, Liu Yang, Zhenggang Zhu, Jun Zhang

**Affiliations:** ^1^Department of Surgery, Ruijin Hospital, Shanghai Jiaotong University School of Medicine, No. 197 Ruijin er Road, Shanghai 200025, China; ^2^Shanghai Institute of Digestive Surgery, Ruijin Hospital, Shanghai Jiaotong University School of Medicine, No. 197 Ruijin er Road, Shanghai 200025, China

## Abstract

*Objective.* To evaluate the efficacy and safety of docetaxel plus oxaliplatin and capecitabine (DOX) in the first line treatment of advanced gastric adenocarcinoma. *Methods.* A total of 37 patients were enrolled into this study, and they received DOX regimen (docetaxel 75 mg/m^2^ and oxaliplatin 130 mg/m^2^ intravenous infusion on day 1, and capecitabine 1000 mg/m^2^ orally twice daily on d1–14); treatment was repeated every 3 weeks. *Results.* All 37 patients were assessable for evaluation. The numbers of patients with complete response (CR), partial responses (PR), stable disease (SD), and progressive disease (PD) were 1, 10, 23, and 3, respectively. The objective response rate (ORR) was 29.7%, with the disease control rate (DCR) of 91.9%. Median progression-free survival (mPFS) and overall survival (mOS) were 197 days and 364 days, respectively. The most common grade 3/4 toxicities were hematological toxicities. The most common grade 3/4 nonhematological toxicities were fatigue, nausea, vomiting, anorexia, diarrhea, and hand-foot syndrome. *Conclusion.* The DOX regimen demonstrated a promising efficacy as the first line regimen in treating advanced gastric cancer patients with good performance status, the toxicities were tolerated and controllable. Large-scale clinical observation is necessary to get further evidence.

## 1. Background

Gastric cancer is the second most common cause of cancer-related deaths worldwide. The outcome of advanced gastric cancer patients is poor, and the median survival time of untreated patients is less than 6 months [1]. A number of randomized clinical trials have established the role of chemotherapy in the treatment of patients with advanced gastric cancer. Compared with those in best support care (BSC) group, the median survival time in chemotherapy groups was longer (7.5–12 months versus 3–5 months). The improvement of median overall survival after chemotherapy was significant in three of the four previously reported studies [2–5]. For many years, 5-fluorouracil plus cisplatin has been considered as the backbone doublet regimen. In some countries, anthracyclin is added to form triplet regimen, such as epirubicin, cisplatin, and continuous-infusion 5-FU (ECF).

During the past decade, several new agents have emerged, including taxanes (paclitaxel, docetaxel), topoisomerase-I inhibitor irinotecan, and third-generation platinum derivative oxaliplatin, which provided more effective and better tolerated regimens in treating this life-threatening disease. 

Although taxanes share a number of pharmacologic characteristics and similar mechanisms of action (tubulin stabilization and cell cycle arrest), they are distinctly different. Docetaxel demonstrated greater affinity for tubulin, longer plasma half-life, and intracellular retention time than paclitaxel. In addition, the taxanes exhibit different profiles of drug resistance [6–10]. Docetaxel has shown activity against gastric cancer either as single agent or in combination with other agents. The results of the V325 trial confirmed the survival benefit and acceptable toxicities of adding docetaxel to cisplatin and fluorouracil in treating advanced or metastatic gastric cancer [11]. Based on the results of this trial, docetaxel was approved by the FDA in 2006 in combination with 5-FU and cisplatin for advanced or metastatic gastric adenocarcinoma.

Oral fluoropyrimidine agents, such as capecitabine and S-1, may provide an alternative to standard infusional 5-FU. Cunningham et al. [12] addressed the results of randomized phase III trial REAL-2. It was a 2 plus 2 factorial design based on the standard European triplet regimen ECF. The overall survival of EOX group, which contains two new cytotoxic agents (oxaliplatin and capecitabine), reached 11.2 months, and significantly longer than those in other groups. The results of REAL-2 study indicated that oxaliplatin was more noninferior than cisplatin, and capecitabine was more noninferior than infusional 5-FU in the first line treatment of advanced and metastatic gastric or gastroesophageal cancer.

Based upon the results of V-325 and REAL-2 studies, we design this single arm phase II study to evaluate the safety and efficacy of DOX (docetaxel, oxaliplatin, capecitabine) regimen in treating unresectable locally advanced or metastatic gastric cancer.

## 2. Methods

### 2.1. Patients Characteristics

The main eligibility criteria included (1) age 18–75 yrs, (2) histologically proven gastric adenocarcinoma, (3) measurable and/or evaluable targeted lesion according to RECIST criteria (version 1.0), (4) good performance status with the Eastern Cooperative Oncology Group (ECOG) score of 0–2, (5) no prior chemotherapy, and (6) adequate hepatic, renal, and hematological function.

Exclusion criteria included (1) concomitant cancers (except melanoma, skin cancer and carcinoma in situ of cervix), (2) neuropathy, brain or leptomeningeal involvement, (3) uncontrolled significant comorbid conditions, (4) patient could not comprehend the purpose of the study and could not comply with the protocol, and (5) women who were pregnant or breastfeeding. The Ethics Committee at Ruijin Hospital, affiliated to Shanghai Jiaotong University School of Medicine, approved the study and written informed consent was obtained.

### 2.2. Treatments

Docetaxel 75 mg/m^2^ was give intravenous infusion for 2 hours on day 1; oxaliplatin 130 mg/m^2^ was also intravenous infusion for 2 hours on day 1, and capecitabine 1000 mg/m^2^ were orally intaked twice daily on d1–14. The regimen was repeated every 3 weeks. The dosage of docetaxel was referred from TAX 325 trial, the dosages of oxaliplatin and capecitabine were referred from REAL-2 and ML17032 trials. Only those who completed at least 2 cycles were assessed efficacy. Patients who finished all 8 cycles of treatment without disease progression were turned into follow-up period. 

### 2.3. Evaluation and Outcomes

Before entering the study, all patients received physical examination, full blood count, and serum chemistry analyses. Chest X-ray, ECG, upper gastrointestinal endoscopies, abdominal computer tomographic (CT) scans, and other appropriate procedures were also performed as needed. During treatment, full blood count and serum chemistry analyses were conducted before each cycle, and X-ray and CT scans were conducted every two cycles. After the treatment completed, patients were receiving these evaluations every 2 months. PFS (progression-free survival) was defined from the day of enrollment to first evidence of disease progression or death occurring within 12 weeks of the last assessable tumor assessment. OS (overall survival) was defined from the date of enrollment to death from any cause. DCR (disease control rate) was defined as follows: complete response (CR) + partial response (PR) + stable disease (SD)/all enrolled patients. Responses were evaluated according to RECIST criteria (version 1.0). Toxicities were graded according to the National Cancer Institute of Canada Common Toxicity Criteria (version 1.0). The primary endpoint of this study was response rates (RR). The secondary endpoints were PFS, OS, DCR, and the toxicity profile. Patients were considered assessable for response if they received at least two cycles of chemotherapy. Safety analyses included all treated patients and involved the analysis of treatment-emergent adverse events. 

### 2.4. Statistical Analysis

The statistical analysis was carried out using SPSS software (version 13.0; SPSS, Chicago, IL, USA). Descriptive statistics were used for safety evaluation. ORR and DCR and their two-sided 95% confidence interval (CI) were calculated. PFS and OS were estimated using Kaplan-Meier method and their medians along with two-sided 95% CIs were calculated.

## 3. Results 

### 3.1. Patients and Treatment Characteristics

A total of 37 late stage gastric cancer patients were enrolled from April 2007 to April 2013. The demographics and baseline characteristics of these patients are presented in Table 1. The median age of these patients was 48 (range 27–72). All patients had good performance status and with ECOG performance status score of 0. Twenty-five patients were confirmed pathologically as poorly differentiated (including signet ring and mucinous carcinoma), 2 patients were moderately differentiated, and the other 10 patients were confirmed as well-differentiated adenocarcinoma. Thirty-one patients had multimetastatic lesions, and the other 6 had locally advanced diseases. 

### 3.2. Overall Response and Survival

**Figure 1:**
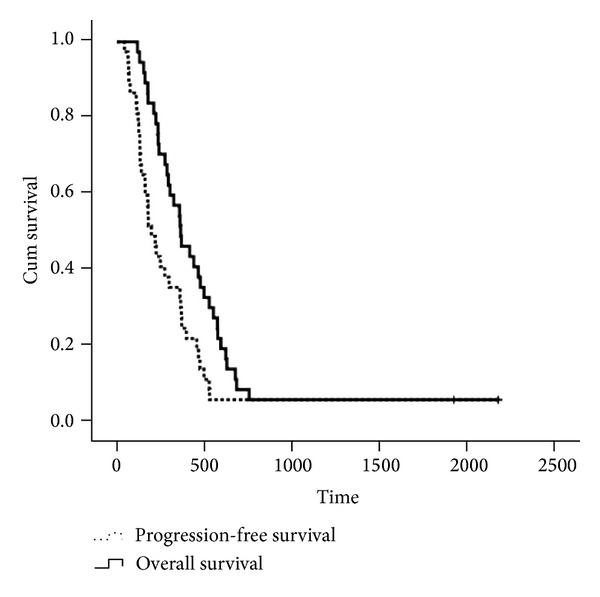
Kaplan-Meier curves of progression-free survival (PFS, 197 days) and overall survival (OS, 364 days) of DOX regimen.

The median number of treatment cycles was six, ranging from one to eight with the total cycles of 216. According to the RECIST criteria (version 1.0), CR was observed in one patient, while PR was observed in 10 patients, SD was in 23 patients, and PD in 3 patients. The ORR was 29.7% (95% CI, 15%–44.4%), while DCR was 91.9% (95% CI, 83.9%–99%). Median progression-free survival (mPFS) and overall survival (mOS) time were 197 days (95% CI, 138–255 days) and 364 days (95% CI, 255–472 days), respectively (Figure 1).

### 3.3. Toxicities

The most common grade 3/4 toxicities were hematological toxicities. The percentages of patients with grade 3/4 leucopenia, neutropenia, and febrile neutropenia were 37.8%, 37.8%, and 10.8%, respectively. The most common grade 3/4 nonhematological toxicities were fatigue, nausea, vomiting, anorexia, diarrhea, and hand-foot syndrome, with the percentages of 27.0%, 5.4%, 5.4%, 8.1%, 2.7%, and 8.1%, accordingly. No severe liver or renal dysfunction or chemotherapy-related death was observed. Only one patient developed grade 2 peripheral neuropathy (Table 2).

### 3.4. Dose Reduction

Patients who developed grade 4 hematological toxicities or grade 3 nonhematological toxicities received a 20% dosage reduction. Among all enrolled patients, 13 had one time dose reduction (docetaxel and oxalipatin were 80% of the initial dose), and other 3 patients got one dose reduction of capecitabine owing to grade 3 hand-foot syndrome. 

## 4. Discussion

The results of this trial showed that triplet regimen with novel agents demonstrated obvious survival benefit as the first-line treatment of late stage gastric adenocarcinoma. The progression-free survival time was 197 days, and the overall survival time was 364 days, which was significantly longer than those in REAL-2 and V-325 trials (Table 3). The reasons might be as follows. First, compared with DCF group in V-325 trial and EOX group in REAL-2 trial, the percentage of metastatic diseases was lower in our study (96% versus 75.5% versus 83.8%). Second, this trial was a single arm noncontrolled trial, and the number of enrolled patients was limited.

The results of V325 and REAL-2 trials, which indicated high efficacy and well tolerability of triplet regimen as the first line treatment of advanced gastric cancer, were referred to in deciding the dose of each chemotherapy agent. In V-325 trial, patients in DCF group were assigned to receive once every 3 weeks docetaxel 75 mg/m^2^ (day 1) plus cisplatin 75 mg/m^2^ (day 1), and fluorouracil 750 mg/m^2^/d continuous infusion (days 1 to 5) [11]. In REAL-2 trial, on day 1 of every 3-week cycle, patients in all study groups received an intravenous bolus of epirubicin (at a dose of 50 mg/m^2^); cisplatin (60 mg/m^2^) was given intravenously with hydration in the ECF and ECX groups, and oxaliplatin (130 mg/m^2^) was administered intravenously during a 2-hour period in the EOF and EOX groups [12]. In addition, patients enrolled in ML17032 trial received cisplatin (80 mg/m^2^ day 1) plus capecitabine (1000 mg/m^2^, days 1–14) or 5-FU (800 mg/m^2^/day by continuous infusion, days 1–5) [13]. The dose of each chemotherapy in this study was referred from the abovementioned large-scale clinical trials.

Similar as those in the other trials, the majority of dose-related grade 3-4 toxicities of docetaxel observed in this trial were hematological toxicities. Of all these 37 patients, the percentage of patients who experienced grade 3/4 leucopenia, neutropenia, and febrile neutropenia were 37.8%, 37.8%, and 10.8%, respectively. With G-CSF support, the hematological toxicities were well controlled and tolerable. No dose cumulative toxicities and chemotherapy-related deaths were observed in this study.

Oxaliplatin is the third-generation platinum-based drug, and has been widely used in the treatment of colorectal carcinoma. Compared with cisplatin, the advantages of oxaliplatin were less renal toxicity and sensorineural hearing loss, as well as no need for hydration. The results of REAL-2 trial demonstrated that oxaliplatin was more noninferior than cisplatin as the first line treatment of metastatic gastric cancer, while combining with fluoropyrimidine and anthracyclin. The results of our study also confirmed the efficacy of this agent as the first line treatment of late-stage gastric cancer, with low incidence of neurotoxicity. 

Either bolus injection or continuous infusion through a central venous access device (CVAD) was used as intravenous administrative routes for fluorouracil. Bolus injection might lead to more toxicities, especially heart toxicity. Yet, continuous infusion was associated with an increased risk of infection and thrombosis as well as the implantation of inconvenient delivery system. Capecitabine is an oral thymidine phosphorylase (TP)-activated fluoropyrimidine. It is an orally administered systemic prodrug of 5′-deoxy-5-fluorouridine (5′-DFUR), which is converted to 5-fluorouracil. It was rationally designed to generate 5-FU preferentially at the tumor site, and it was activated in tumor tissue by a three-step enzymatic conversion culminating with TP. This high tumor selectivity is achieved through exploitation of the significantly higher activity of TP in tumor tissue compared with healthy tissue. Capecitabine mimics continuous infusion 5-FU and potentially offers a more convenient alternative to intravenous infusion. Some cytotoxic agents, such as taxanes, oxaliplatin, and cyclophosphamide, are known to up-regulate TP activity in tumor cells, offering potential synergistic action. Several powerful clinical trials, such as REAL-2, ML17032, and ToGA, have elucidated the efficacy of capecitabine in chemonaïve gastric cancer patients [14].

The SPIRITS trial evaluated S-1 plus cisplatin versus S-1 alone in the first-line treatment of advanced gastric cancer. A total of 148 patients were assigned to S-1 plus cisplatin group and 150 patients were assigned to S-1 monotherapy group. Median overall survival was significantly longer in S-1 plus cisplatin group than that in S-1 group (13.0 months versus 11.0 months; HR = 0.77; 95% CI, 0.61–0.98; *P* = 0.04). Progression-free survival was also significantly longer in doublet group (6.0 months versus 4.0 months; *P* < 0.0001) [15]. In another phase II study of docetaxel and S-1 combination therapy for advanced or recurrent gastric cancer, an overall response rate of 56.3% (95% confidence interval (95% CI), 38–66%) was observed and the tumor control rate (complete response, partial response, and stable disease) was 93.8% (95% CI, 83–98%). Median overall survival was 14.3 months (95% CI, 10.7–20.3 months) and median time to tumor progression was 7.3 months (95% CI, 4.3–10.0 months) [16]. All these two trials demonstrated survival benefits of S-1 in advanced gastric cancer, which holds the promise of becoming a backbone agent in the first-line regimen in treating advanced gastric cancer. 

The results of AVAGAST [17] and REAL-3 [18] trials failed to demonstrated survival benefit of adding bevacizumab or panitumumab to standard chemotherapy in the first line of gastric cancer. HER-2 enrichment designed study ToGA trial showed the efficacy and safety of trastuzumab in the first line treatment of HER-2 positive advanced gastric cancer. Patients with HER-2 immunohistochemistry (IHC)+++ or IHC++/FISH positive (15~20%) can benefit from this targeted agent, with mOS of 16.0 months. For those with HER-2 negative, the optional chemotherapeutic strategies, including the selection of agents and regimen, the lines to be addressed, and the potential role of target identification, should be further investigated. 

The results of our study demonstrated a promising good response, survival benefit, and well tolerance of triplet regimen, including docetaxel, oxaliplatin, and oral fluoropyrimidine capecitabine, in the first line treatment of late stage gastric cancer patients with good performance status. The shortage of this study is single arm, limited patient number, and lack of biomarker exploration. The precise efficacy of this regimen, and the subgroup of gastric cancer patients who benefit more from this regimen need to be investigated by a large-scale study.

## Figures and Tables

**Table 1:** Patients characteristics (*n* = 37).

Characteristics	No.
Sex	
Male	23
Female	14
Age (yr)	
Median	48
Range	27–72
ECOG	
0-1	37
2	0
Extent of disease	
Locally advanced	6
Metastasis	31
Pathology	
Moderate	2
Poor	25
Other	10
Organs involved	
Liver	12
Lung	2
Bone	3
Pelvic cavity	11
Virchow's LN	3
Previous treatment	
Exploratory laparotomy	8
Gastrectomy	7
Chemotherapy	0

**Table 2:** Grade 3-4 toxicities of DOX regimen.

Toxicity	*n* (%)
Hematological toxicity	
Leukopenia	14 (37.8)
Neutropenia	14 (37.8)
Febrile neutropenia	4 (10.8)
Nonhematological toxicity	
Gastrointestinal toxicity	
Nausea	2 (5.4)
Vomiting	2 (5.4)
Anorexia	3 (8.1)
Diarrhea	1 (2.7)
Neurosensory toxicity	1 (2.7)
Hepatic	1 (2.7)
Other	
Fatigue	10 (27.0)
Hand-foot syndrome	3 (8.1)

**Table 3:** Cross-trial comparison.

	V325		REAL-2				
	DCF	CF	ECF	ECX	EOF	EOX	DOX
Median age (yr)	55	55	65	64	61	62	48
Range (yr)	26~79	25~76	22~83	25~82	33~78	25~80	27~72
Metastasis (%)	96	97	79.5	76.8	77	75.7	83.8
One least dose reduction (%)	41.2	36.2	/	/	/	/	43.2
OS (m)	9.2	8.6	9.9	9.9	9.3	11.2	11.9
PFS (m)	5.6	3.7	6.2	6.7	6.5	7	6.5
RR (%)	37	25	40.7	46.4	42.4	47.9	29.7
CR (%)	2	1	4.1	4.2	2.6	3.9	2.7
PR (%)	35	24	36.6	42.2	39.8	44	27
SD (%)	30	31	/	/	/	/	62.2
Adverse events							
Leukopenia	65	31	/	/	/	/	37.8
Neutropenia	82	57	41.7	51.1	29.9	27.6	37.8
Fabric neutropenia	29	12	9.3	6.7	8.5	7.8	10.8
Hand-foot syndrome	/	/	4.3	10.3	2.7	3.1	8.1
Nausea	14	17	10.2	7.7	13.8	11.4	5.4
Vomit	14	17					5.4
Diarrhea	19	8	2.6	5.1	10.7	11.9	2.7
Fatigue	19	14	16	15.5	12.9	24.9	27
